# Machine Learning for Predicting Risk of Early Dropout in a Recovery Program for Opioid Use Disorder

**DOI:** 10.3390/healthcare10020223

**Published:** 2022-01-25

**Authors:** Assaf Gottlieb, Andrea Yatsco, Christine Bakos-Block, James R. Langabeer, Tiffany Champagne-Langabeer

**Affiliations:** 1 School of Biomedical Informatics, University of Texas Health Science Center at Houston, 7000 Fannin St., Houston, TX 77030, USA; Assaf.Gottlieb@uth.tmc.edu (A.G.); andrea.j.yatsco@uth.tmc.edu (A.Y.); christine.bakosblock@uth.tmc.edu (C.B.-B.); james.r.langabeer@uth.tmc.edu (J.R.L.); 2 McGovern Medical School, University of Texas Health Science Center at Houston, 6431 Fannin St., Houston, TX 77030, USA

**Keywords:** opioid use disorder, machine learning, predictive modeling, treatment, substance use disorder

## Abstract

Background: An increase in opioid use has led to an opioid crisis during the last decade, leading to declarations of a public health emergency. In response to this call, the Houston Emergency Opioid Engagement System (HEROES) was established and created an emergency access pathway into long-term recovery for individuals with an opioid use disorder. A major contributor to the success of the program is retention of the enrolled individuals in the program. Methods: We have identified an increase in dropout from the program after 90 and 120 days. Based on more than 700 program participants, we developed a machine learning approach to predict the individualized risk for dropping out of the program. Results: Our model achieved sensitivity of 0.81 and specificity of 0.65 for dropout at 90 days and improved the performance to sensitivity of 0.86 and specificity of 0.66 for 120 days. Additionally, we identified individual risk factors for dropout, including previous overdose and relapse and improvement in reported quality of life. Conclusions: Our informatics approach provides insight into an area where programs may allocate additional resources in order to retain high-risk individuals and increase the chances of success in recovery.

## 1. Introduction

According to the U.S. Department of Health and Human Services (HHS), an estimated 10.1 million people aged 12 or older used opioids in the United States in 2019, and 1.6 million people had a diagnosed opioid use disorder (OUD) [1]. Of these, over 48,000 people died from synthetic opioid-involved overdoses. In 2017, the HHS declared a public health emergency and announced a five-point strategy to combat the opioid crisis.

The Houston Emergency Opioid Engagement System (HEROES) was established in 2018 and created rapid access to a long-term recovery pathway for individuals with OUD. The program includes access to board-certified physicians for initiation of an approved medication for opioid use disorder (buprenorphine), plus additional comprehensive care that incorporates peer recovery coaching, individual and group counseling, and navigation to other services and resources based upon need, including recovery housing, employment, and support for justice-involved individuals [2].

As has been previously demonstrated, longer treatment retention is associated with a greater likelihood of abstinence from opioids [3]. However, early dropout is common in substance use treatment settings and may lead to poorer outcomes relative to those completing a full course of treatment [4]. Protective factors against recurrence of use and overdose are higher for individuals who remain active in treatment [5]. According to the principles of treatment outlined by the National Institute on Drug Abuse, remaining in treatment for an adequate amount of time is critical, and a minimum of 90 days is recommended [6].

Previous studies have focused on identifying factors that reduce dropout rate. Two studies of 78 and 202 participants focused on short-term dropout (3 and 4 months, respectively) [4,7]. Both identified young age as the leading factor underlying dropout rate, with the second study also identifying opioid use in the first month. A recent study focused on multiple dimensions, including demographic and psychosocial variables, and discovered that patients with greater stress levels and a sustained history of emotional abuse were more likely to drop out of treatment [8]. Finally, two longer-term studies found that younger age, minority ethnicity, and unemployment status were also associated with poor retention [9,10]. The studies mentioned calculated single-factor statistics and not overall risk.

Machine learning methods for developing prediction models are data-driven and growing in their application to substance use treatment analysis [11]. For example, two machine learning methods have attempted to take the prediction task from individual factors to multi-factorial models based on administrative claims data. Acion et al. compared different machine learning methods for predicting completed treatments as an indication of treatment success [12], but their study did not take into account the time in which the treatment was discontinued. Hasan et al. focused on buprenorphine treatment and predicted buprenorphine discontinuation within one year [13]. However, this study relied only on available drug prescription, resulting in low performance. Furthermore, prediction for discontinuation after a year limits the ability to detect problems early and possibly intervene.

Therefore, we decided to utilize machine learning methods to improve retention rates in our program and to inform the literature on treatment retention. Leveraging this approach may offer program administrators and clinicians the opportunity to divert resources and engage participants at the highest risk for drop out early in the program. We introduced a machine learning model to a retrospective database of over 700 enrolled patients to identify individuals at higher risk of dropping from the program after 90 and after 120 days. The benefit of our approach over previous methods is that we are using a richer set of information on each participant in the program, including demographic, clinical, behavioral/mental health, and extensive history about prior substance use and criminal activity. Furthermore, as the program is ongoing, we can directly implement an intervention program to retain those at high risk for early dropout (after 90 and 120 days). Thus, our encouraging model performance in detecting the set of risk factors in these high-risk individuals provides a tool for our program and like-programs across the U.S. for retention and ultimately for improving recovery for individuals suffering with OUD, as they are retained longer in the program.

## 2. Materials and Methods

### 2.1. Data

**Figure 1:**
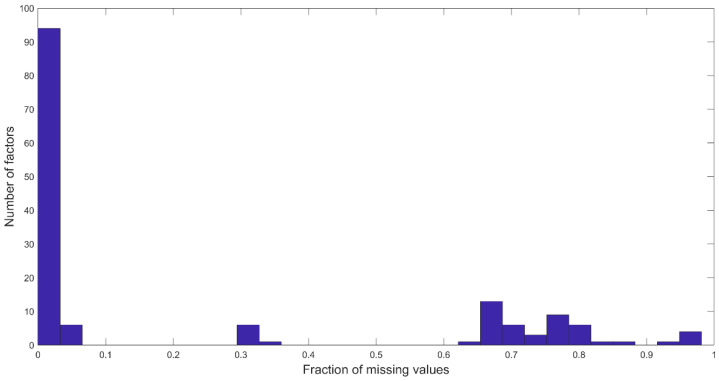
Histogram of the fraction of missing values per factor. Factors with over 50% missing values were removed.

As of May 2021 (here forth termed “index date”), the program database included 715 individuals that had joined more than 90 days before the index date and 691 individuals that had joined more than 120 days prior to the index date. The data are collected through self-reported questionnaires and through a health evaluation by a nurse practitioner or physician assistant. Data are stored in a Research Electronic Data Capture (REDCap) project database [14] (see Supplement, S1: BioPsychosocial_HEROES). Out of these individuals, 105 (15%) and 165 (24%) had dropped out of the program within less than 90 days and 120 days, respectively. Categorical factors (i.e., factors with more than two discrete values) were converted into dummy variables, resulting in 163 factors, including 10 continuous value factors and 153 dichotomous factors (binary) that describe these individuals. We further removed 55 factors with more than 50% missing values (Figure 1). We imputed each of the remaining 108 factors, including 1 continuous factor (age) and 107 dichotomous factors, using the average over the available information for each factor, which translates to imputing with the fraction of positive values in each factor.

### 2.2. Prediction Scheme

We tested several machine learning classifiers, including logistic regression, radial basis support vector machine, random forest (with 100 trees, and the default parameters in Matlab’s TreeBagger implementation), and five different variants of ensemble based on boosting algorithms based on decision trees, including Adaptive boosting (AdaBoost), Gentle Boost, LogitBoost, Robust Boosting, and totally corrective boosting [15,16,17,18]. We used 10-fold cross-validation and used the specificity and sensitivity metrics to evaluate our predictions, where for the boosting algorithm, we applied a nested cross-validation scheme that uses training, testing, and validation sets for hyperparameter tuning with Matlab’s automatic boosting algorithm, which uses Bayesian optimization. Due to the large imbalance between the positive (those who dropped out) and the negative (those who were retained) groups, we sampled from the negative group the same number of individuals as in the positive group and ran the cross-validation procedure, repeating the sub-subsampling process five times with different random sub-samples to verify the robustness of the results. Results provided are averaged over the five independent repetitions. All analyses were conducted using Matlab R2019a.

### 2.3. Factor Ranking

We estimated the predictor importance by permutation of out-of-bag predictor observations in the random forest classifier model. Additionally, we verified that these ranking were consistent with the association in statistical tests, where we used Mann–Whitney U test between the retained and dropped out groups for continuous factors and Fisher exact test for dichotomous factors. Significant factors with *q*-values (i.e., *p*-values adjusted for Benjamini-Hochberg (BH) false discovery rate) of .05 were selected.

## 3. Results

### 3.1. Machine Learning Framework to Identify Program Dropout

**Figure 2:**
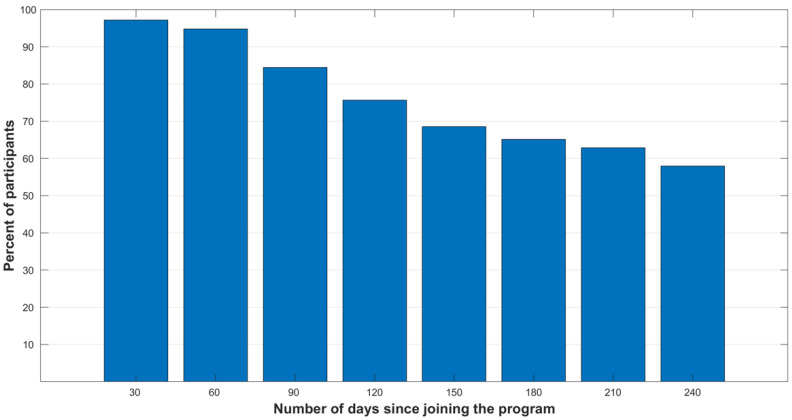
Program retention rates at 30-day time intervals. Y-axis displays the fraction of individuals retained at each time point since joining the program.

We evaluated the dropout rate in increments of 30 days (Figure 2), identifying that the dropout became substantial at 90- and 120-days periods reaching 15% (90 days) and 24% (120 days). Thus, we focused on predicting the characteristics of the patients likely to drop out of the program after 90 and 120 days.

**Figure 3:**
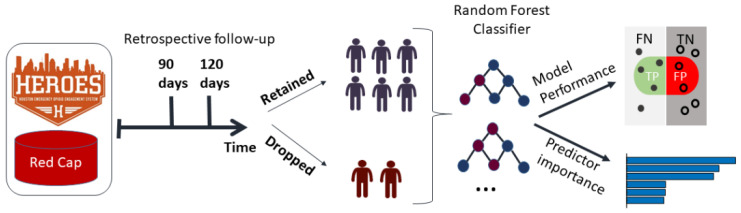
Schematic of the data analysis pipeline. Information on the individuals that are enrolled in the program and longitudinal information is recorded in a REDCap database. A retrospective analysis generated a prediction model based on information collected prior to the index dates at 90 and 120 days.

We tested several machine learning classifiers to predict dropout (Methods, Table 1), selecting random forest as our optimal classifier (see Figure 3 for data analysis plan). Random forest had slightly better overall performance tradeoff than the rest of the classifiers, having the highest sensitivity (recall) with a comparable specificity to other classifiers (Table 1). We considered higher sensitivity more important than the prediction outcome, since having a low number of false negatives (fewer individuals who dropped out that were missed by the classifier) would be more critical for retention than possibly investing more resources in higher number of false positives (individuals we marked as high risk for dropout but would not drop out) with the current lower specificity. Thus, we chose random forest as our selected classifier. The classifier obtained sensitivity of 0.81 ± 0.02 and specificity of 0.65 ± 0.05 for the 90-day threshold and sensitivity of 0.86 ± 0.03 and specificity of 0.66 ± 0.02 for the 120-day threshold.

### 3.2. Risk Factors Associated with Program Dropout

We next identified factors associated with dropout using ranked predictor importance in the classification model using out-of-bag predictor observations technique and verified using statistical tests on each independent factor (Methods). There were 27 factors that passed the false discovery rate of 0.05 at both time points (Table S1).

Three factors were consistently ranked high in both the 90- and 120-day check dates, identified both by statistical test and predictor importance in the prediction model. These features include whether the individual have overdosed in the past (Benjamini-Hochberg false discovery rate (FDR)-adjusted, *p* < e^−15^), improvement in quality of life (FDR-adjusted, *p* < 4e^−15^), and whether they relapsed since joining the program ((FDR-adjusted, *p* < 3e^−6^), see Table 2. Interestingly, we did not observe significant differences in the ages of the individuals who dropped out and those who were retained as a previously published factor.

The top factor was whether the individual had overdosed in the past. Individuals who dropped out had 3.2 to 4.8 times higher incidence of past overdosing (i.e., the number of individuals with past dosing was 3.2 to 4.8 times higher in the dropout group, adjusted by group size). The second top factor was improvement in quality of life. More than 94% of the individuals who dropped out have reported improvement in their quality of life while only 64% (90 days)–59% (120 days) in the retained group reported improved quality of life. Finally, the group of individuals who dropped out had a 1.8–2 times higher incidence of reported relapses since joining the program.

## 4. Discussion

We have presented a model for predicting early dropout from a long-term recovery program for opioid use disorder at 90 and 120 days. Our model obtained good sensitivity and specificity and enables the identification of the top contributing factors. Overall, analyses of treatment dropout rates were low for program participants, with 85% active and engaged in treatment at 90 days and 76% active at 120 days post enrollment. Compared to a recent systematic and meta-analysis review of dropout rates that reports average dropout rates for targeted opioid treatment programs (39%) and target heroin treatment programs (26%), overall retention was high for participants in the analyzed program model [19]. While retention in the program was high overall, there were some distinguishable differences between those who dropped out of treatment versus those who remained in treatment.

Some variables were significantly related to retention probability. The top contributing factor was whether the individual had overdosed in the past. Individuals who dropped out of the program had overdosed in the past more than those who were retained, suggesting that individuals with an extensive history of substance use may need additional support maintaining engagement in an outpatient program. A recent study found individuals who experienced a non-fatal overdose were at increased risk of successive overdose and were more likely to engage in polysubstance use [20,21]. Moreover, polysubstance use may contribute to lifetime prevalence of overdose. Additional research from the National Epidemiologic Survey on Alcohol and Related Conditions III found that 98% of individuals who reported opioid use reported using other substances [22]. The correlation between opioid use, polysubstance use, and lifetime prevalence of overdose is well established by research, further supporting comprehensive services, including treatment for polysubstance use.

The second top factor was improvement in quality of life. More than 94% of those who dropped out reported an improvement in their quality of life, while only 64% (90 days)–59% (120 days) in the retained group reported improvements, per the quality-of-life survey. We estimate that individuals who had experienced improvements in their quality of life may hold the perception of doing well and feel that they do not need the same level of treatment to maintain their recovery, or they may have transitioned to longer-term programs with less intensity. This type of non-adherence to treatment or medication protocols when one is feeling better is not isolated to OUD. Similar studies focused on patients with depression have found that individuals stop taking their medications when they are feeling better [23]. While most research supports improvements in reported quality of life during substance abuse treatment, there is no consensus on the exact relationship between quality of life and treatment retention or dropout. Some research found quality of life increased early on in treatment from baseline, then decreased or diminished after leaving treatment [24,25,26,27,28]. Although research supports quality of life as an important factor in sustained abstinence, there are myriad of factors related to quality of life, and no standardized instrument to measure it among those with OUD [29,30].

Finally, individuals who dropped out had higher incidence of relapses since joining the program, which is likely an indication they needed increased accountability, additional or varied support, or would benefit from a referral to a higher level of care [31]. OUD is a chronic and relapsing condition, with relapse rates surpassing 90% [32,33]. Individuals with multiple psychosocial problems and instability are often hard to engage and retain in treatment [34]. Although MOUD is shown to reduce opioid-related mortality, some individuals receiving MOUD continue to use opioids and are at risk for a fatal overdose [35]. Another study found that individuals with an overdose-related ED encounter in the previous year were least likely to be abstinent from opioid use at baseline and more likely to use illicit benzodiazepines while enrolled in treatment [21]. Research on predictors of relapse has found that psychological factors, such as history of trauma and depression, and biological factors, such as cortisol levels and adrenal sensitivity, are associated with increased risk of relapse [36]. These data support an extensive approach to treatment that addresses biopsychosocial factors associated with relapse among those receiving MOUD.

Recovery from addiction is multifactorial and may take multiple attempts for an individual. Treatment that includes MOUD, behavioral health, and social support is widely accepted as the most effective treatment; yet, a considerable number of individuals receiving treatment perpetually relapse and often overdose, suggesting treatment resistance in some patients with OUD [37]. There are other treatment-resistant disorders and diseases, including mood disorders, cancer, and hypertension, which are widely accepted and supported by science; therefore, the idea of treatment-resistant OUD may be plausible and calls for further research [38,39,40]. We also found that individuals with past prescription opioid use, past legal issues, or a history of benzodiazepines were more likely to drop out at 90 days (Supplementary Table S1). Our findings support other research that has found use of benzodiazepines and current and past legal issues associated with increased dropout [10]. These types of activities may signal a lack of “readiness” for recovery or the ability to create change at the time of treatment [41,42].

Previous studies have identified younger age, cocaine and heroin use, lower doses of methadone, criminal activity/incarceration, and negative attitudes to methadone maintenance treatment as associated factors [43,44]. While age was not significant in our analysis, cocaine and heroin use and incarceration were statistically significant (*p* < 0.008), but did not reach the top ranked features listed in Table 2. Methadone use was not recorded in our data.

While we obtained good prediction performance, there could be factors that were not collected on these individuals that affect their choice to drop out of the program. One limitation of our model is that we use only structured factors. However, the program also collects free text information on participants that could potentially be converted to additional features that will enhance the model for future studies. Overall, our findings are consistent with previous research on treatment retention and support further exploration of treatment-resistant opioid use disorder.

The findings from this research support the concept of using a tiered risk approach, ranging from high to low risk of early dropout. A predictive model may be developed with a greater understanding of the factors that influence patient participation choices. The model allows for a system of classification; therefore, resources can be allocated efficiently, and treatment plans can be tailored to the individual. For example, participants in the highest risk group may receive more intensive follow-up from peer recovery coaches multiple times per week and might be required to attend additional group counseling and one-on-one counseling. Higher risk groups may also be required to submit urine drug tests on a regular basis to confirm abstinence from illicit substances, while participants in the lowest risk group may receive follow up from a peer recovery coach less often and be required to attend group counseling and one-on-one counseling every few weeks. By customizing the treatment and care provided to participants within each group, it becomes possible to provide participants with the greatest needs with the attention and resources required.

## 5. Conclusions

In this research, we analyzed variables for persistence in treatment for a cohort of individuals engaging in a program for OUD. We developed a data-driven prediction model using machine learning techniques and found that individuals with prior medication for OUD and multiple relapses may be considered higher risk and more likely to drop out of the program. Further qualitative research is needed to determine the optimal timing of interventions and the varied services needed for individuals to persist in treatment.

## Figures and Tables

**Table 1:** Performance of different machine learning classifiers on the prediction of dropout.

	90 Days		120 Days	
Method	Specificity	Sensitivity	Specificity	Sensitivity
Logistic Regression	0.22 ± 0.3	0.9 ± 0.13	0.44 ± 0.27	0.81 ± 0.12
Radial Basis Support Vector Machines	0.54 ± 0.02	0.63 ± 0.04	0.57 ± 0.03	0.66 ± 0.03
AdaBoost	0.62 ± 0.05	0.82 ± 0.07	0.66 ± 0.01	0.83 ± 0.03
Gentle Boost	0.63 ± 0.05	0.81 ± 0.02	0.67 ± 0.04	0.79 ± 0.03
Logit Boost	0.64 ± 0.05	0.79 ± 0.04	0.68 ± 0.02	0.81 ± 0.04
Robust Boost	0.62 ± 0.06	0.79 ± 0.02	0.69 ± 0.06	0.8 ± 0.05
Total Boost	0.61 ± 0.03	0.81 ± 0.02	0.66 ± 0.03	0.84 ± 0.02
Random Forest	0.65 ± 0.05	0.81 ± 0.02	0.66 ± 0.02	0.86 ± 0.03

**Table 2:** Top contributing factors associated with individuals who dropped out at 90 and 120 days. Individuals with missing data are excluded.

Factor	Individuals Who Dropped 90 Days (%)	Individuals Retained 90 Days (%)	Individuals Who Dropped 120 Days (%)	Individuals Retained 120 Days (%)	FDR-Adjusted *p*-Values (90 Days, 120 Days)
Have you overdosed?	58 (56%)	91 (17%)	84 (52.2%)	49 (11.0%)	e^−15^, 2e^−26^
QoL improvement	100 (95%)	347 (64%)	155 (93.9%)	269 (59.0%)	4e^−15^, 4e^−25^
Have you relapsed since joining?	58 (55%)	169 (31%)	90 (54.5%)	126 (27.6%)	3e^−6^, 2e^−11^

## Data Availability

The datasets generated and/or analyzed during the current study are not publicly available due to them containing information that could compromise research participant privacy/consent.

## Supplementary Materials

The following supporting information can be downloaded at: https://www.mdpi.com/article/10.3390/healthcare10020223/s1, S1: BioPsychosocial_HEROES. Table S1. (This is the intake form used to collect participant data upon entry into the treatment program).

## Abbreviations

AdaBoost	Adaptive Boosting
AUC	Area Under the Curve
FDR	False Discovery Rate
HEROES	Houston Emergency Opioid Engagement System
OUD	Opioid Use Disorder
