# Supplementary material for: Machine Learning for Predicting Risk of Early Dropout in a Recovery Program for Opioid Use Disorder
Source: Healthcare (Basel). 2022 Jan 25;10(2):223. doi: 10.3390/healthcare10020223 (PMC8871589; doi:10.3390/healthcare10020223)
Supplement: Supplementary file 1 [file healthcare-10-00223-s001.zip › healthcare-1566378-supplementary.pdf]

Table S1: Factors significantly associated with early dropout. Factors are ordered by p-value for the association at 90 days.

Description	Adjusted p-value 90d	Adjusted p-value 120d	Model weight 90d	Model weight 120d
Quality of life improvement	4.E-15	4.E-25	0.64	0.88
Have relapsed since joining the program	3.E-06	2.E-11	0.37	0.67
Have used prescription oxycodone	5.E-05	3.E-04	0.36	0.22
Past legal problems	6.E-05	8.E-05	0.24	0.13
Substance use history of benzodiazepines	1.E-04	3.E-05	0.28	0.11
Are you a veteran?	1.E-04	8.E-06	0.18	0.27
Female	7.E-04	1.E-05	0.19	0.33
First time taking medications for opioid use disorder	1.E-03	6.E-03	0.18	0.32
Housing status: Other	2.E-03	4.E-06	0.30	0.53
Prior use of barbiturates	3.E-03	2.E-04	0.16	0.12
Substance use history of hallucinogens	8.E-03	4.E-06	0.13	0.21
Substance use history of non Rx opiates	1.E-02	4.E-05	0.15	0.19
Substance use history of cocaine	1.E-02	1.E-05	0.11	0.20
Prior use of non Rx suboxone	2.E-02	2.E-03	0.08	0.10
Taken medications for opioid use disorder from inpatient services	2.E-02	1.E-02	0.14	0.14
Substance use history of alcohol	2.E-02	6.E-05	0.11	0.16
Prior use of cocaine	2.E-02	2.E-05	0.08	0.18
Housing status: Homeless	2.E-02	4.E-03	0.37	0.29
Substance use history of Marijuana	2.E-02	4.E-05	0.13	0.13
Prior use of nicotine or tobacco	2.E-02	3.E-05	0.12	0.14
Drug of choice? (choice=Other substances)	2.E-02	7.E-03	0.04	0.00
Prior arrest for drug charges	2.E-02	9.E-03	0.02	0.09
Consider themselves as neither religious nor spiritual	2.E-02	4.E-02	0.07	-0.02
Substance use history of heroin	2.E-02	2.E-04	0.04	0.11
Prior arrest for parole or probation violations	2.E-02	3.E-02	0.03	0.06
Ever taken medications for opioid use disorder in outpatient services	4.E-02	1.E-05	0.23	0.39
Consider yourself to be spiritual	4.E-02	1.E-04	0.06	0.15
